# Structural and Functional Insights into Small, Glutamine-Rich, Tetratricopeptide Repeat Protein Alpha

**DOI:** 10.3389/fmolb.2015.00071

**Published:** 2015-12-18

**Authors:** Joanna D. Roberts, Arjun Thapaliya, Santiago Martínez-Lumbreras, Ewelina M. Krysztofinska, Rivka L. Isaacson

**Affiliations:** Department of Chemistry, King's College LondonLondon, UK

**Keywords:** SGTA, quality control, protein sorting, structural biology, biophysical techniques, cochaperones

## Abstract

The small glutamine-rich, tetratricopeptide repeat-containing protein alpha (SGTA) is an emerging player in the quality control of secretory and membrane proteins mislocalized to the cytosol, with established roles in tail-anchored (TA) membrane protein biogenesis. SGTA consists of three structural domains with individual functions, an N-terminal dimerization domain that assists protein sorting pathways, a central tetratricopeptide repeat (TPR) domain that mediates interactions with heat-shock proteins, proteasomal, and hormonal receptors, and viral proteins, and a C-terminal glutamine rich region that binds hydrophobic substrates. SGTA has been linked to viral lifecycles and hormone receptor signaling, with implications in the pathogenesis of various disease states. Thus far, a range of biophysical techniques have been employed to characterize SGTA structure in some detail, and to investigate its interactions with binding partners in different biological contexts. A complete description of SGTA structure, together with further investigation into its function as a co-chaperone involved quality control, could provide us with useful insights into its role in maintaining cellular proteostasis, and broaden our understanding of mechanisms underlying associated pathologies. This review describes how some structural features of SGTA have been elucidated, and what this has uncovered about its cellular functions. A brief background on the structure and function of SGTA is given, highlighting its importance to biomedicine and related fields. The current level of knowledge and what remains to be understood about the structure and function of SGTA is summarized, discussing the potential direction of future research.

## Introduction

Protein quality control networks have evolved to deal with the burden of transiently unfolded and terminally misfolded proteins a cell has to face, that result from inefficiencies in protein folding, assembly and targeting (Wickner et al., 1999). The small glutamine-rich, tetratricopeptide repeat-containing protein alpha (SGTA) is a co-chaperone involved in a specific branch of the global cellular quality control network that determines the fate of secretory and membrane proteins that mislocalize to the cytosol (Leznicki and High, 2012; Wunderley et al., 2014). SGTA recognizes surface exposed regions of hydrophobicity on newly synthesized tail-anchored (TA) proteins and mislocalized proteins (MLPs), and shields them from the aqueous cytosol thus preventing them from misfolding, forming undesirable protein-protein interactions, and from aggregation. Upon stabilization of these exposed hydrophobic regions, the subsequent fate of the proteins is determined, which may result in refolding to their native conformation, targeting to their correct destination or being marked for degradation (Leznicki and High, 2012; Leznicki et al., 2013, 2015; Wunderley et al., 2014). This review collates and integrates the current literature surrounding SGTA structure, function and interactions.

SGTA was first identified in complex with viral proteins (Callahan et al., 1998; Cziepluch et al., 1998) and has emerged as a key regulator in macromolecular quality control, in addition to its established roles in the biogenesis of TA membrane proteins, and proposed roles in hormone receptor signaling (Leznicki and High, 2012; Leznicki et al., 2013; Philp et al., 2013; Wunderley et al., 2014). SGTA has been found to localize predominantly to the cytoplasm, however its presence in the nucleus has also been detected (Philp et al., 2013). Due to its ubiquitous expression in human tissues, and interactions with many different proteins including HIV-1 encoded proteins Vpu and Gag, hormone receptors and myostatin, SGTA is associated with both health and disease (Callahan et al., 1998; Kordes et al., 1998; Schantl et al., 2003; Wang et al., 2003).

## Structural overview of SGTA

**Figure 1:**
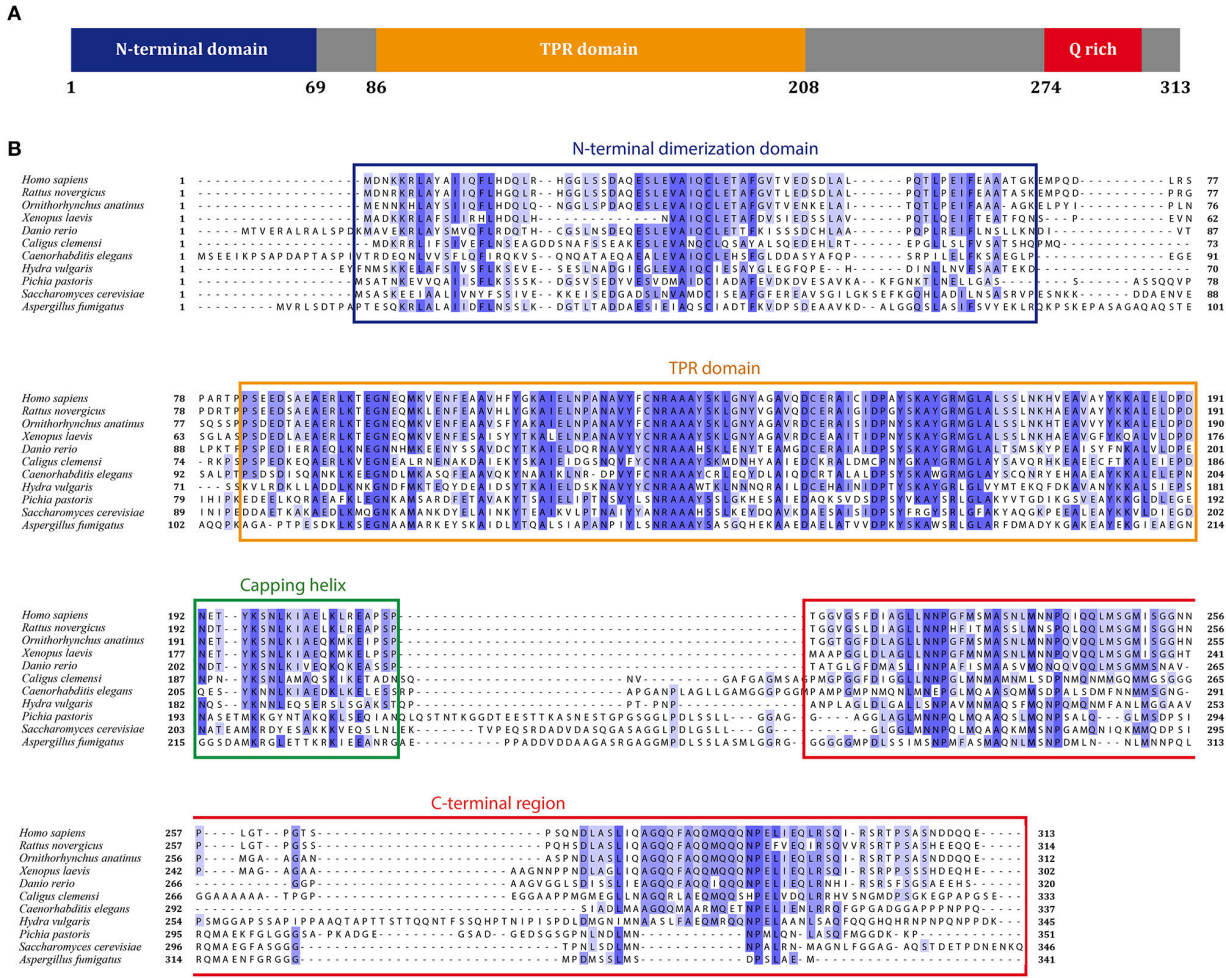
**SGTA sequence alignment. (A)** Full length SGTA is made up of 313 residues: an N-terminal homodimerization domain (residues 1–69) followed by a 14-residue linker, three TPR repeats with a distinct capping helix (residues 86–208) and a C-terminal domain (residues 211–313) including a Q-rich region (residues 274–313). **(B)** Sequences of SGTA and its homologs across the phyla have been aligned, and domains annotated with reference to the human SGTA sequence. *C. elegans, D. rerio*, and *A. fumigatus* appear to have longer N-terminal extensions compared to both mammalian and yeast equivalents. The central TPR repeats are the most conserved, however the TPR capping helix (helix 7) exhibits lower conservation in fungal species than in metazoans. The glutamine-rich region, present in the C-terminal domain, also exhibits greater similarity in metazoans, with the presence of NNP repeats conserved across all phyla.

Human SGTA is a 34 kDa protein made up of 313 amino acids that is ubiquitously expressed across all tissue types to varying levels (Cziepluch et al., 1998; Kordes et al., 1998). It assembles as a homodimer, with each chain comprising three structural domains (Figure 1A): an N-terminal dimerization domain (residues 1–69) followed by a flexible linker of around 14 residues, a central tetratricopeptide repeat (TPR) domain (residues 86–208) and a C-terminal domain (residues 211–313) which includes a 39 amino acid glutamine rich region (amino acids 274–313). SGTA is highly conserved in different eukaryotes, and in particular shows significant sequence homology amongst metazoans (Figure 1B). The N-terminal and TPR domain boundaries are clearly identifiable from sequence alignments, with the central TPR repeats being the most conserved. The C-terminal domain incorporates stretches of glutamine residues, the positions of which are conserved in higher eukaryotes. Additionally, the glutamine-rich region exhibits strong similarity in metazoans, with the presence of C-terminal NNP repeats that are conserved across all phyla.

SGTA is unique in being the only TPR-containing steroid receptor co-chaperone known to form a homodimer (Buchanan et al., 2007). This dimerization ability was initially attributed to the TPR domain, which is capable of inter- and intra-molecular interactions in other proteins (Hirano et al., 1990; Cziepluch et al., 1998). However, it was observed in a brain-specific isoform (β-SGT), and later in SGTA, that homo-dimerization is mediated by the N-terminus (Tobaben et al., 2003; Liou and Wang, 2005). In addition, the dimer is thought to be elongated, with the three domains adjacent to each other (Liou and Wang, 2005; Worrall et al., 2008; Darby et al., 2014). Evidence for this is gleaned from the reported hydrodynamic radius of its *C. elegans* equivalent (Sgt2) which has been observed to be larger than that of a globular protein (Worrall et al., 2008). Small angle X-ray scattering (SAXS) studies concur with an elongated arrangement of the yeast Sgt2 dimer devoid of its C-terminal domain, with full length Sgt2 showing characteristics of a partially folded protein (Chartron et al., 2011). Apart from the above, there is no structural information of domain organization in the context of the full-length protein and the lack of conservation of the linker between the N-terminal domain and the TPR, together with its length (10–15 amino acids) suggests the presence of a flexible connection between these two domains. Apart from the N-terminal dimerization, no further evidence of intra- or inter-domain contacts have been observed thus far in fully assembled SGTA dimers.

Contrary to our understanding of the full-length dimer, there have been several high-resolution structural studies on excised SGTA domains. The structure of SGTA TPR domain was determined by X-ray crystallography and was the first of its three domains to be structurally elucidated (Dutta and Tan, 2008). In addition, the structure of its N-terminal domain has been extensively characterized, which has provided insights into its role in TA protein insertion pathways, together with details of its association with other key effectors involved in MLP quality control pathways, such as the BAG6 complex (Chartron et al., 2011; Simon et al., 2013a,b; Darby et al., 2014). However, the C-terminal region of SGTA is yet to be structurally characterized, hence the nature and extent of its hydrophobic substrate-binding site, along with mechanistic details pertaining to its interactions with TA proteins and MLPs, remain poorly understood. A summary of all currently solved SGTA and Sgt2 structures is shown in Table 1.

**Table 1:** **A summary of structurally characterized domains of SGTA/Sgt2**.

**Protein/Species**		**Domain**	**Residues**	**Technique**	**PDB**
SGTA	*Homo sapiens*	N-terminal	3–54	X-ray crystallography	4GOD
			1–69	Solution NMR spectroscopy	4CPG
		TPR	80–210	X-ray crystallography	2VYI
Sgt2	*Saccharomyces cerevisiae*	N-terminal	1–78	Solution NMR spectroscopy	4ASV
			2–72	Solution NMR spectroscopy	2LXB
Sgt2	*Aspergillus fumigatus*	TPR	104–254	X-ray crystallography	3SZ7

## N-terminal dimerization domain

**Figure 2:**
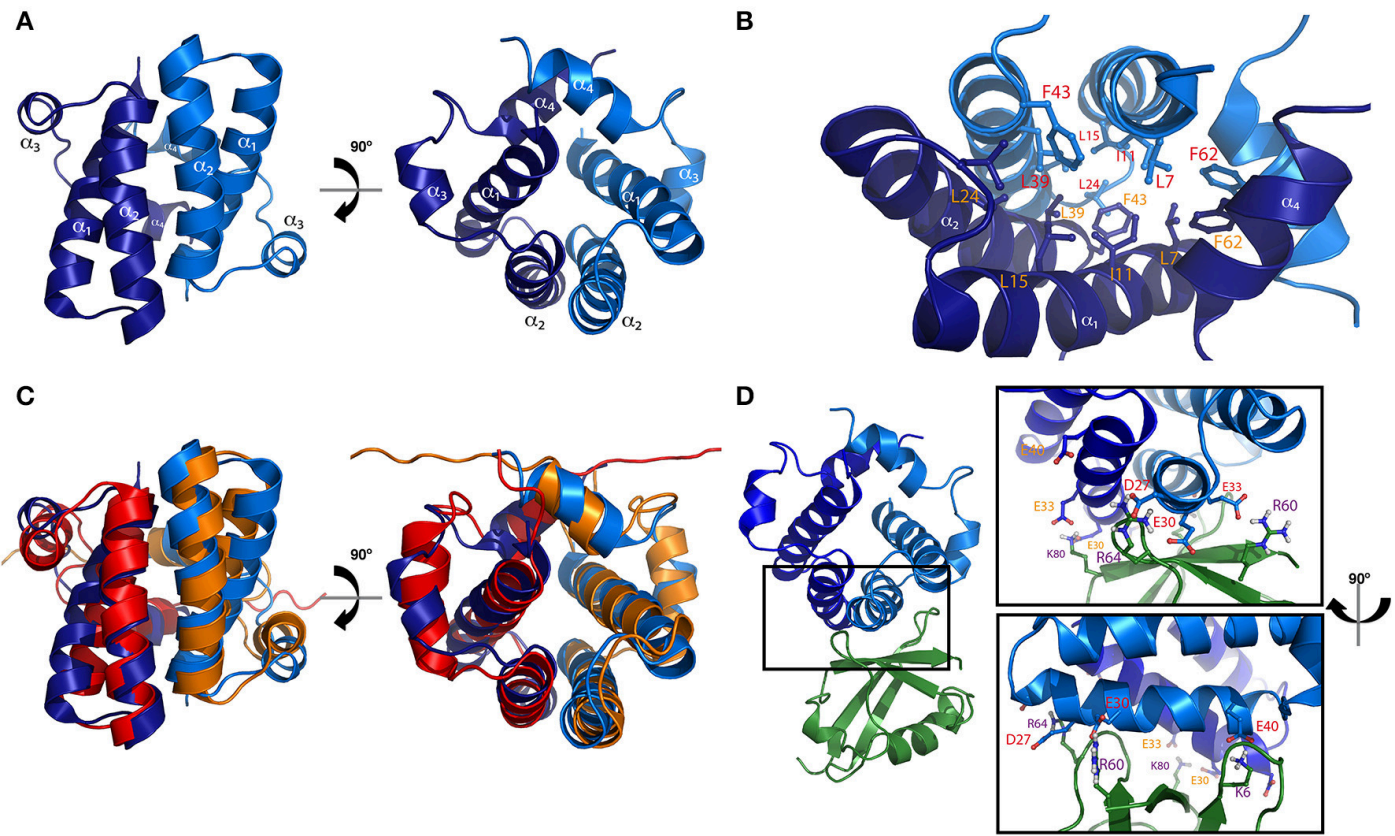
**N-terminal homodimerization domain of SGTA. (A)** Two orthogonal views representing the solution NMR structure of the N-terminal domain of mammalian SGTA (PDB accession code 4CPG) are shown in blue indicating the four alpha helices present in each protomer (α_1_–α_4_). **(B)** Hydrophobic residues present in the inner region of the mammalian N-terminal dimer, forming a tight core, are shown as sticks. **(C)** Overlay of solution NMR structures of N-terminal dimers from mammalian SGTA (blue) and yeast Sgt2 (orange-red, PDB accession code 4ASV), are shown as two orthogonal views. An RMSD of 2.41Å is observed between these two structures. **(D)** The ubiquitin binding domain (UBD) of the N-terminal dimer of mammalian SGTA (in blue) shown in complex with a cognate UBL domain (in green), with residues exhibiting a strong electrostatic component at the binding interface shown as sticks (inset; Darby et al., 2014).

The N-terminal homodimerization domain of SGTA is formed of four α-helices in each constituent protomer (α_1_ residues 3–21, α_2_ residues 26–43, α_3_ residues 47–52, and α_4_ residues 58–67) connected by short loops, which arrange into a unique helical fold (Figure 2A). The dimer interface is made up of hydrophobic residues that form a tight interaction, resembling the core of a globular protein (Figure 2B; Simon et al., 2013b; Darby et al., 2014). This characteristic fold is conserved in the N-terminal domain of the yeast homolog Sgt2, presenting an RMSD of 2.41Å between the two structures (Darby et al., 2014; Figure 2C).

The N-terminal domain of SGTA is responsible for its interactions with the ubiquitin-like (UBL) domains present in the BAG6 complex (Simon et al., 2013b; Darby et al., 2014). The N-terminal domain dimer presents a negatively charged surface, arising primarily from residues present in the second alpha helix (Asp27, Glu30, Glu33, and Glu40), that recognize a single UBL domain. The UBL exhibits a positively charged region (up to 5 arginine or lysine residues) at which binding is mediated through electrostatic interactions. In addition, there are some apolar residues in both proteins that are buried in the interface creating further stabilization of the complex through hydrophobic interactions (Figure 2D; in SGTA mainly Cys38 and Val34, and in UBLs two conserved positions of leucine/isoleucine; Chartron et al., 2012). The association with UBLs breaks the symmetry of the N-terminal dimer, and the stoichiometry of the resulting complex (i.e., SGTA dimer binds to one UBL domain) was confirmed using several techniques such as solution nuclear magnetic resonance (NMR), isothermal titration calorimetry (ITC), and microscale thermophoresis (MST; Simon et al., 2013a,b; Darby et al., 2014).

## TPR domain

TPR domains are versatile in both structure and function, they are found widely in multi-subunit protein assemblies and consist of between 3 and 16 tandem repeats, with each repeat made of 34 amino acid residues forming a helix-turn-helix motif (Das et al., 1998; D'Andrea and Regan, 2003). They mediate specific protein-protein interactions, exploiting their structural variability to define their functionality and a minimum of three tandem repeats is required for their function (Blatch and Lässle, 1999). Normally, a solubility/stability helix is present at the C-terminus of most TPRs, which may be an essential feature of the domain (Das et al., 1998). The TPR consensus sequence is variable but reveals a pattern of large and small hydrophobic amino acid residues, which are highly conserved at key positions vital for both structure and function. Other positions are less conserved and substitution within a class of amino acids with similar properties is tolerated, thus, consistency in size and overall hydrophobicity can be observed (Sikorski et al., 1990; Blatch and Lässle, 1999).

**Figure 3:**
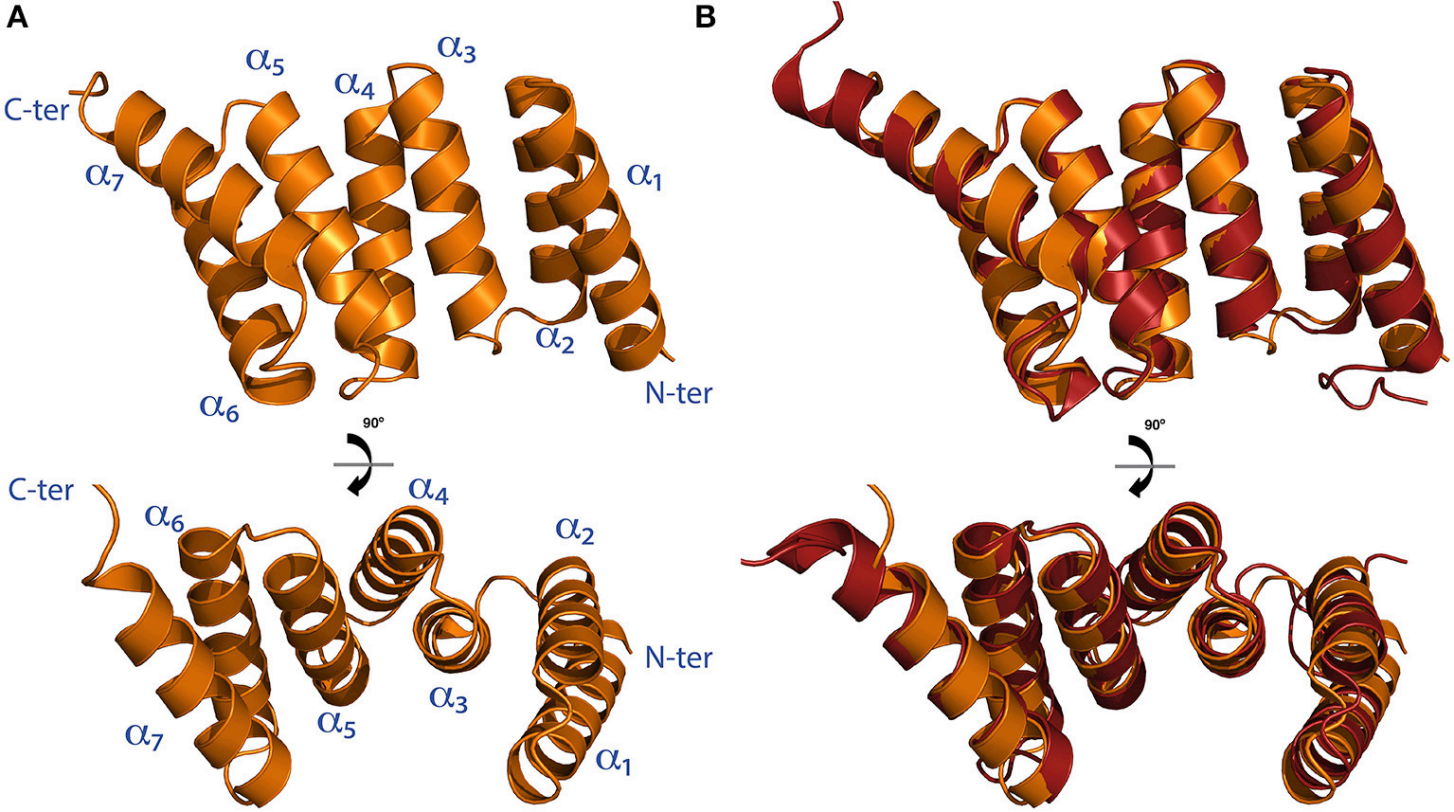
**The TPR domain of SGTA. (A)** The X-ray crystal structure of the TPR domain of human SGTA is shown as two orthogonal views (PDB accession code 2VYI). The three helix-turn-helix repeats formed by helices α_1_/α_2_,α_3_/α_4_, and α_5_/α_6_ can be observed in the structure, followed by a capping helix (α_7_), to form a right handed superhelical assembly (Dutta and Tan, 2008). **(B)** TPR domain crystal structure of human SGTA (shown in orange) superimposed with that of *A. fumigatus* Sgt2 (shown in red; PDB accession code 3SZ7). The TPR structure of the fungal homolog presents a longer capping helix oriented at a 10° angular increment when compared to the equivalent region of human SGTA-TPR (Chartron et al., 2011).

The structure of the TPR domain of human SGTA was solved in 2008 by X-ray crystallography (Figure 3A; Dutta and Tan, 2008). It consists of three TPR motifs arranged in tandem, each formed by a pair of α-helices folded in an antiparallel fashion, in which the three TPR repeats are almost structurally identical. This is followed by a C-terminal capping helix (helix 7) that packs against the second helix of the third TPR motif. The seven helices arrange into a right-handed superhelical structure with a concave surface lined by helices α1, α3, α5, and α7 as in other TPR domains (D'Andrea and Regan, 2003; Dutta and Tan, 2008). The structure of the TPR domain of Sgt2 from a fungal homolog *A. fumigatus* has also been determined by X-ray crystallography revealing a similar architecture compared to the TPR domain of human SGTA (38% sequence identity, 60% similarity and a Cα RMSD value of 1.2Å). The main differences between these two structures can be observed at their C-terminal regions, in which the fungal homolog has a capping helix (α7) five residues longer, and is positioned at a 10° angular increment relative to the α6 helix when compared to the human TPR crystal structure (Figure 3B; Chartron et al., 2011).

In comparison to known TPR domain structures, despite their low sequence similarity, the fold of the human SGTA TPR domain was found to be highly similar to two other co-chaperone TPR domains, namely the carboxyl-terminus of Hsp70 interacting protein (CHIP) and Hsp70/90 organizing protein HOP (Dutta and Tan, 2008). Both structures bind to EEVD, the C-terminal binding motif of Hsp70 and Hsp90 chaperones, an interaction that occurs through highly conserved key residues (Scheufler et al., 2000; Zhang et al., 2005). Superposition of SGTA-TPR with CHIP-TPR bound to EEVD revealed that essential binding residues are also present in SGTA-TPR, suggesting that human SGTA may bind to Hsp70/90 in a similar fashion to CHIP and HOP. Subsequently, two different studies confirmed SGTA-TPR binding to Hsp70 and Hsp90 soon after the structure was solved, and both studies suggested binding to occur through the EEVD motif (Liu et al., 1999; Liou and Wang, 2005). These binding interactions of SGTA through its TPR motif appear to provide a physical link with ATP-dependent molecular chaperones which may attest to its co-chaperone functionality. Apart from serving as a chaperone binding platform, the TPR domain also interacts with several proteins such as hormone receptors, viral proteins and myostatin (Wang et al., 2003). Interactions with viral proteins have been based on pulldowns, Co-IP and yeast two hybrid assays, localizing the TPR domain of SGTA as the binding platform for Vpu and Gag from HIV (Handley et al., 2001), ORF7a from SARS-CoV (Fielding et al., 2006), NS1 from parvovirus H-1 (Cziepluch et al., 1998) and for the human endogenous retrovirus protein HERV-K (HML-2; Hanke et al., 2013). While the interaction of ORF7a from SARS-CoV has been mapped to the second TPR motif (Fielding et al., 2006), structural details of protein-protein interactions with other viral proteins remain unclear. Similarly, in the case of TPR domain interactions with hormone receptors, apart from the first TPR motif interacting with the growth hormone receptor (Schantl et al., 2003), very little is known about binding modes with these proteins. In many cases, the possibility of binding indirectly through other linking proteins cannot be ruled out and further work is necessary to characterize the interactions.

## C-terminal domain

The C-terminal domain of SGTA includes a glutamine-rich region, which consists of a stretch of 39 amino acids punctuated by 12 glutamine residues (Cziepluch et al., 1998; Liou and Wang, 2005). The functional significance of the C-terminal domain was investigated by yeast two-hybrid (Y2H) screening, sampling different sized fragments of SGTA that were expressed and screened against a library of proteins. Full length SGTA and fragments containing the C-terminal domain were found to interact with polypeptide fragments containing six or more consecutive non-polar residues. SGTA with its C-terminus removed did not interact with the hydrophobic fragments showing that the C-terminus was necessary for interaction with hydrophobic substrates (Liou and Wang, 2005). This capability of the C-terminal domain facilitates interactions of SGTA in the BAG6 quality control cycle of MLPs, its ability to shield TMD helices of TA proteins facilitating their post-translational membrane integration, and its interaction with the cysteine-string protein, a chaperone implicated in neurotransmitter release (Tobaben et al., 2003; Wang et al., 2010). The cysteine string protein was also identified by Y2H screening as a binding partner for the C-terminal domain (Tobaben et al., 2001). Its site of interaction was mapped to a string of 14 cysteine residues embedded within a hydrophobic region; removal of this region disrupted its interaction with SGTA (Tobaben et al., 2003). SGTA C-terminal domain has also been shown to interact with exposed hydrophobic regions on myostatin (together with the third TPR repeat), and on an integral membrane protein of the rat type 1 glucose transporter (Wang et al., 2003; Liou and Wang, 2005).

The complete lack of high-resolution structural information on this region of SGTA means molecular details pertaining to its interactions with hydrophobic substrates remain unclear. There are many outstanding questions with regard to this vital, yet poorly understood, substrate-binding domain of SGTA. These include mechanisms of TA-protein recognition and sorting, and the binding and release of MLPs determining their ultimate fate.

## SGTA in protein homeostasis and quality control

**Figure 4:**
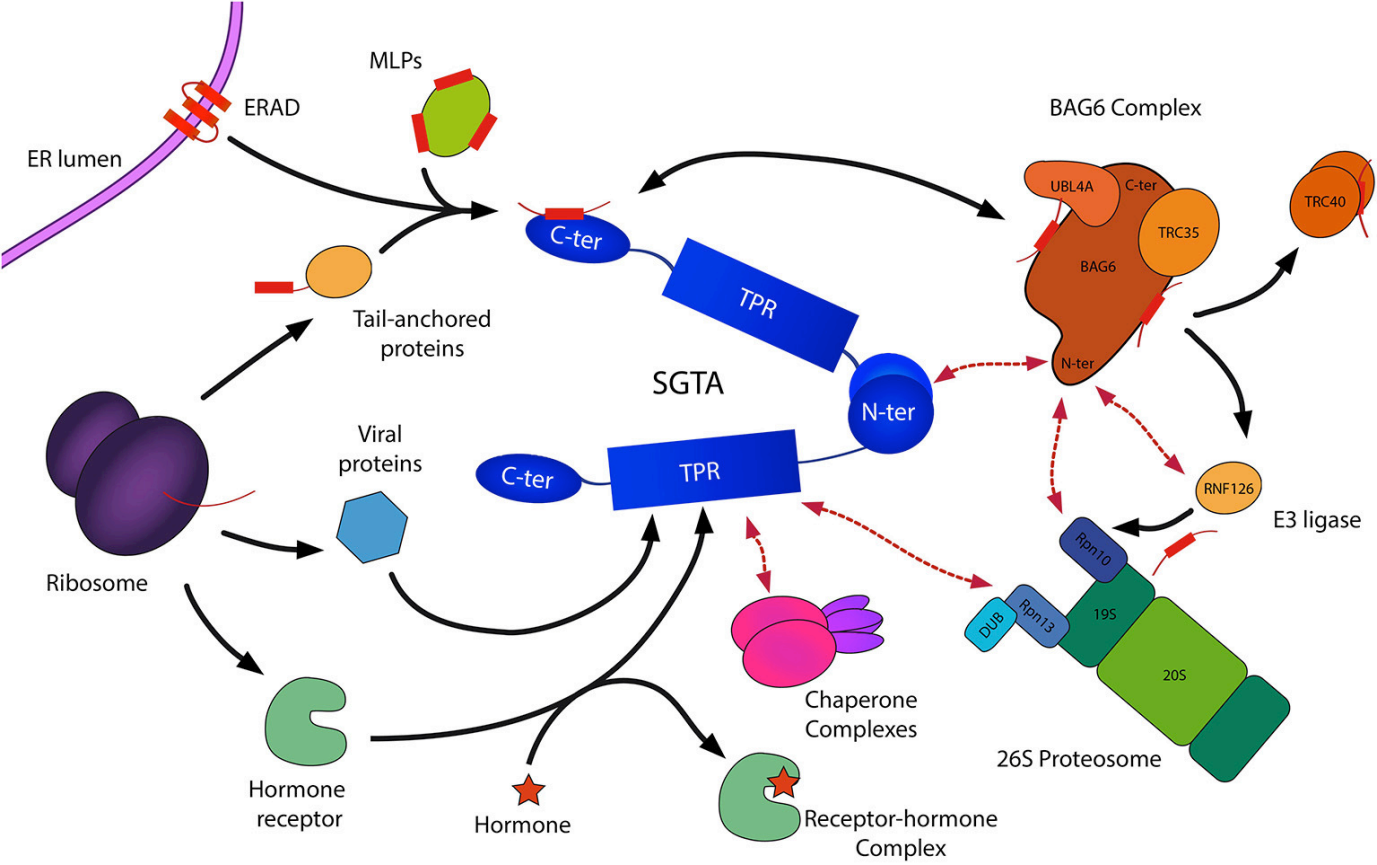
**Schematic representation of SGTA's biological roles**. SGTA is involved in the quality control of hydrophobic substrates (MLPs, ERAD substrates), a process mediated by its C-terminal domain in collaboration with the BAG6 complex (Payapilly and High, 2014; Wunderley et al., 2014). The heterotrimeric BAG6 complex is composed of BAG6, TRC35, and UBL4A proteins, and interacts with SGTA via its UBLs (Darby et al., 2014). Hydrophobic substrates bound to the BAG6 complex are ubiquitinated by the actions of the E3 ligase RNF126, thus targeted for proteasomal degradation (Rodrigo-Brenni et al., 2014). SGTA interacts with the RPN13 subunit of the 19S regulatory particle of the proteasome through its TPR domain, which has led to the proposal of an SGTA/BAG6 cycle operating at the proteasome (Leznicki et al., 2015). Additionally, SGTA's role extends to the shielding of exposed hydrophobic regions on TA proteins facilitating their post-translational integration into the ER (Hegde and Keenan, 2011). This enables the handover of TA proteins to the downstream TRC40 targeting complex (Stefanovic and Hegde, 2007). Furthermore, SGTA has been implicated in hormone receptor signaling (Schantl et al., 2003; Buchanan et al., 2007; Paul et al., 2014), and has been associated with viral lifecyles (Callahan et al., 1998; Handley et al., 2001; Walczak et al., 2014). SGTA's interactions with Hsp70/Hsp90 chaperones (Liou and Wang, 2005) via its TPR domain may provide substrate access to additional branches of the global cellular quality control network.

SGTA interacts with a range of chaperone networks and pathways through its central TPR domain (Figure 4; Liou and Wang, 2005). However, as mentioned earlier, SGTA's role in the quality control of hydrophobic substrates is first mediated by its C-terminal domain. The ability of SGTA to recognize and shield surface exposed regions of hydrophobicity, termed “degrons,” on MLPs prevents substrate aggregation and promotes normal cellular proteostasis (Chartron et al., 2011; Kawahara et al., 2013; Wunderley et al., 2014). This aspect of SGTA function is carried out in concert with the BAG6 complex.

The heterotrimeric BAG6 complex is composed of BAG6 (BCL2-associated athanogene), TRC35 (transmembrane recognition complex 35) and UBL4A (ubiquitin-like protein 4A) proteins, and plays an integral role in maintaining cellular protein homeostasis through its involvement in different quality control pathways (Mariappan et al., 2010; Payapilly and High, 2014). As SGTA can bind to both UBLs in the BAG6 complex (BAG6_UBL and UBL4A_UBL) through its homodimerization domain, this provides a direct link between the two quality control factors. In this SGTA/BAG6 cycle, SGTA directs MLPs toward deubiquitination and hence stabilization while BAG6 directs MLPs toward polyubiquitination and proteasomal degradation. BAG6 recognizes MLPs and recruits an E3 ubiquitin ligase, RNF126, which facilitates substrate access to the ubiquitin-proteasome system (UPS; Rodrigo-Brenni et al., 2014). SGTA is capable of reversing ubiquitination initiated by the BAG6 complex, thus rescuing proteins from degradation via an as yet unidentified deubiquitinating enzyme (DUB; Leznicki and High, 2012). Therefore, it has been suggested that SGTA/BAG6 dependent cycles of substrate ubiquitination and deubiquitination may be responsible for the kinetic partitioning of correctly folded precursors and terminally misfolded substrates (Wunderley et al., 2014; Leznicki et al., 2015). Thus, SGTA is intricately associated with the quality control function of BAG6 in deciding the fate of hydrophobic substrates, either promoting them toward maturation leading to correct insertion into the ER, or marking them for degradation (Hessa et al., 2011; Leznicki and High, 2012; Leznicki et al., 2013; Wunderley et al., 2014).

SGTA and the BAG6 complex also assist the endoplasmic reticulum-associated degradation pathway (ERAD), in which terminally misfolded proteins are ubiquitinated and retrotranslocated out of the ER into the cytosol leading to their proteasomal degradation (Xu et al., 2012; Payapilly and High, 2014). Once retrotranslocated into the cytosol, ERAD substrates are known to associate with BAG6, which maintains them in a soluble state preventing their aggregation (Payapilly and High, 2014). Recent studies indicate a role of SGTA in modulating the fate of ERAD substrates, in which SGTA overexpression has been shown to delay their proteosomal degradation (Wunderley et al., 2014).

## SGTA acts at the proteasome

Recent discoveries indicate that SGTA associates with the 19S regulatory particle of the 26S proteasome through a direct interaction between its TPR domain and the C-terminal domain of the intrinsic proteasomal ubiquitin receptor RPN13 (Leznicki et al., 2015). Furthermore, it is known that the BAG6 subunit of the heterotrimeric BAG6 complex interacts with the intrinsic proteasomal ubiquitin receptor RPN10 (Kikukawa et al., 2005; Minami et al., 2010). On this basis, a dynamic quality control cycle operating at the 19S regulatory particle of the proteasome has been proposed, capable of determining the fate of MLPs targeted for degradation (Leznicki et al., 2015). This model suggests that an SGTA/BAG6 cycle operating at the proteasome may regulate access of MLPs to the proteolytic core, allowing substrates several rescue attempts before being committed to proteasomal degradation. This potential rescue pathway is in turn aided by the proteasome associated deubiquitinase UCH37/UCHL5, that also collaborates with RPN13 (Bhattacharyya et al., 2014), providing a mechanism for multiple cycles of ubiquitination and deubiquitination through the concerted actions of SGTA and the heterotrimeric BAG6 complex. This proposed pathway could potentially facilitate the rescue of viable substrates from proteasomal degradation.

## SGTA in the biogenesis of tail-anchored membrane proteins

SGTA plays an important role in the post-translational integration of tail-anchored membrane (TA) proteins (Wang et al., 2010; Johnson et al., 2013). TA proteins are a family of membrane proteins characterized by a functional cytoplasmic domain tethered to the lipid bilayer by a single pass transmembrane domain (TMD) helix of moderate hydrophobicity present at their extreme C-terminus, a region which also includes the membrane targeting signal (Hegde and Keenan, 2011). TA proteins constitute around 5% of all human membrane proteins, including SNAREs, ER translocon components and signaling proteins (Simpson et al., 2010). In mammals, these proteins are delivered to the ER membrane by a conserved transmembrane domain recognition complex (TRC) pathway in which SGTA, together with the BAG6 complex, plays a vital role in shielding TMD regions on newly synthesized TA proteins from the aqueous cytosol as they are released by the ribosome. This facilitates TA protein transfer to the dedicated TRC40 (transmembrane domain recognition complex 40) targeting complex before being passed on to membrane receptors for insertion (Stefanovic and Hegde, 2007; Leznicki et al., 2010, 2011; Chartron et al., 2012). In yeast, the equivalent TA insertion pathway is referred to as the guided entry of tail-anchored proteins (GET) pathway. The GET pathway involves handover of TA protein substrates bound to the Sgt2 co-chaperone (the yeast equivalent of SGTA) to the Get3 ATPase, a process mediated by the Get4/Get5 complex. This is followed by subsequent TA-protein release at the ER membrane by the Get1/Get2 receptor complex (Simpson et al., 2010). Thus, SGTA/Sgt2 facilitate the biogenesis of TA proteins by ensuring their correct maturation. Also, as TA protein substrates are more prone to premature ubiquitination, it has been suggested that SGTA dependent deubiquitination of TA proteins provides a mechanism for rescue and subsequent transfer to downstream targeting factors (Leznicki and High, 2012; Wunderley et al., 2014).

## SGTA in the regulation of hormone receptor signaling

SGTA interacts with steroid receptor complexes and signaling pathways including those of the androgen receptor (AR), a nuclear transcription factor (Paul et al., 2014). AR signaling pathways are critical in the pathogenesis of hormone related diseases including prostate cancer (Buchanan et al., 2007), breast cancer and polycystic ovary syndrome, pathologies in which SGTA has been found to be upregulated (Goodarzi et al., 2008; Zhu et al., 2014). With respect to AR signaling, it has been shown that SGTA interacts with the AR hinge region through its TPR domain (Buchanan et al., 2007). Moreover, a member of endogenous retroviruses of the HERV-K (HML-2) family, namely the protein Rec, has been proposed to modulate AR activity through its interaction with SGTA (Hanke et al., 2013). Further investigations into these potential roles of SGTA will be required to delineate its contribution to the AR signaling pathway. In addition, SGTA has been associated with interactions that involve endocytosis of the growth hormone receptor. In order to understand the role of SGTA in hormone receptor recognition, yeast two hybrid and pull-down assays have been performed that localize the interaction of these receptors to the TPR domain, in particular to the first TPR motif in the case of growth hormone receptor (Schantl et al., 2003). It has also been shown that SGTA regulates the activity of glucocorticoid and progesterone receptors (Paul et al., 2014). However, in the absence of molecular details pertaining to direct physical interactions of SGTA with hormone receptors, mechanistic details elucidating relevant pathophysiological states remain poorly understood.

## The role of SGTA in viral infections

SGTA's association with viral infections was first described in the context of SGTA as a binding partner for NS1, a nonstructural protein of parvovirus H-1 essential for viral DNA replication and transcriptional gene expression. This was based on data suggesting SGTA localization in both the cytoplasm and nucleus of rat fibroblasts, potentially implicating SGTA in parvoviral replication and/or gene expression (Cziepluch et al., 1998). Another study identified the association between SGTA and an accessory severe acute respiratory syndrome coronavirus protein 7a (SARS-CoV 7a), with the TPR domain of SGTA being essential for this interaction (Fielding et al., 2006). However, details pertaining to mechanisms of binding, and ways in which SGTA is implicated in the life cycle of these viruses still need to be elucidated.

The function of SGTA in human immunodeficiency virus type 1 (HIV-1) particle release has been studied in some detail (Callahan et al., 1998; Handley et al., 2001). It is known that SGTA is engaged in Vpu mediated enhancement of viral particle release by an interaction with the HIV-1 Gag protein, a viral core protein precursor. SGTA can bind to both Vpu and Gag, and its overexpression in mammalian cell lines transfected with HIV-1 proviral constructs has shown to reduce the efficiency of virus particle release (Callahan et al., 1998). Additionally, it has been proposed that SGTA can support the shuffling of the viral protein Gag to the plasma membrane where it can assembly into HIV-1 virus capsids (Handley et al., 2001). However, *in vivo* association of SGTA and Gag is abolished when Vpu is expressed in the cell. It is also known that Vpu can affect cellular localization of SGTA and Gag proteins (Callahan et al., 1998; Handley et al., 2001). Furthermore, the TPR domain proved to be sufficient for SGTA's interaction with Vpu and Gag albeit with lower efficiency than full length SGTA, therefore potential contributions of N- and C-terminal domains cannot be excluded (Dutta and Tan, 2008). Interestingly, it has also been proposed that SGTA may be involved in promoting viral infections where it can facilitate virus transport from ER to cytosol by assisting in membrane penetration, as recently demonstrated in the case of Simian virus 40 (SV40; Walczak et al., 2014).

## Conclusions and future goals

It is becoming increasingly apparent that SGTA is emerging as a key effector in the cellular quality control of MLPs and also as a protein with wider roles. These include the regulation of protein biogenesis and maturation, modulation of protein degradation, regulation of hormone receptor signaling and viral lifecycles (Figure 4). However, mechanistic details pertaining to most of these roles have yet to be fully understood.

Recent studies combining various biochemical, biophysical and cell biological approaches have revealed much about the role of SGTA in cellular functions as part of the SGTA/BAG6 quality control cycle. However, it is still unclear how this cycle differentiates between different classes of hydrophobic substrates in order to direct them along appropriate downstream pathways. A key milestone in understanding SGTA function will be achieved through a complete molecular understanding of the structural and functional contributions of its C-terminal domain, in particular, a description of how it binds hydrophobic substrates. This will yield insight into SGTA's substrate specificity and how it triages different kinds of hydrophobic substrates, such as TA proteins, MLPs, and ERAD substrates.

It is known that SGTA promotes the deubiquitination of client proteins and hence appears to facilitate their rescue, or at least enables further attempts at promoting the acquisition of a functional fold. This relies on deubiquitinating enzymes (DUBs) that collaborate with components of the SGTA/BAG6 cycle. Further investigation is necessary to identify these DUBs and understand their contribution to this process.

SGTA has long been associated with Hsp70 and Hsp90 chaperones (Liu et al., 1999; Liou and Wang, 2005). It is tempting to speculate that collaboration with these chaperones will enable SGTA substrates access to additional quality control pathways. Thus, elucidation of the precise mechanism whereby SGTA substrates interact with Hsp70/Hsp90 chaperones will improve our understanding of proteostasis networks in which these components operate. Similarly, a detail description of SGTA's role in hormone receptor signaling is essential to understand its regulatory function in hormonally induced disease states. With more details relating to SGTA's role in viral lifecycles emerging (Walczak et al., 2014), extensive biochemical characterization is necessary to arrive at the complete picture.

Solving the structure of full-length SGTA by a variety of methods will go a long way toward understanding its interactions with all the binding partners in macromolecular detail and shed light on the overall mechanical capabilities of the dimer. The piecemeal structures that currently exist for SGTA and BAG6 complex components have been useful first steps in understanding their roles but larger complex structures will add a great deal and represent an urgent goal for researchers in this area. Overall, it is imperative to get an improved understanding of the proteostatic networks in which SGTA operates under physiological conditions, to further our understanding of how these pathways are reconfigured in various disease states. It is hoped that these insights into pathologies will ultimately feed in to the design of therapeutics.

## Funding

We gratefully acknowledge the financial support of the Biotechnology and Biological Sciences Research Council (BBSRC) of the UK. RLI and SM-L are funded by BBSRC grant: BB/L006952/1. AT is funded by BBSRC grant: BB/J014567/1.

### Conflict of interest statement

The authors declare that the research was conducted in the absence of any commercial or financial relationships that could be construed as a potential conflict of interest.
